# Host population structure and species resolution reveal prophage transmission dynamics

**DOI:** 10.1128/mbio.02377-24

**Published:** 2024-09-24

**Authors:** Karen Tenorio-Carnalla, Alejandro Aguilar-Vera, Alfredo J. Hernández-Alvarez, Gamaliel López-Leal, Valeria Mateo-Estrada, Rosa Isela Santamaria, Santiago Castillo-Ramírez

**Affiliations:** 1 Programa de Genómica Evolutiva, Centro de Ciencias Genómicas, Universidad Nacional Autónoma de México, Cuernavaca, Mexico; 2 Unidadad de Administración de Tecnologías de Información, Universidad Nacional Autónoma de México, Cuernavaca, México; 3 Laboratorio de Biología Computacional y Virómica Integrativa, Centro de Investigación en Dinámica Celular, Universidad Autónoma del Estado de Morelos, Cuernavaca, México; Institut Pasteur, Paris, France

**Keywords:** ANI, bacteriophage genetics, population genomics, phage species, prophages, species definition, *Acinetobacter baumannii*

## Abstract

Much knowledge about bacteriophages has been obtained via genomics and metagenomics over the last decades. However, most studies dealing with prophage diversity have rarely conducted phage species delimitation (aspect 1) and have hardly integrated the population structure of the host (aspect 2). Yet, these two aspects are essential in assessing phage diversity. Here, we implemented an operational definition of phage species (clustering at 95% identity, 90% coverage) and integrated the host’s population structure to understand prophage diversity better. Gathering the most extensive data set of *Acinetobacter baumannii* phages (4,152 prophages + 122 virulent phages, distributed in 46 countries in the world), we show that 91% (875 out of 963) of the prophage species have four or fewer prophages per species, and just five prophage species have more than 100 prophages. Most prophage species have a narrow host range and are geographically restricted; yet, very few have a broad host range being well spread in distant lineages of *A. baumannii*. These few broad host range prophage species are not only cosmopolitan but also the most abundant species. We also noted that polylysogens had very divergent prophages, belonging to different prophage species, and prophages can easily be gained and lost within the bacterial lineages. Finally, even with this extensive data set, the prophage diversity has not been fully grasped. Our study highlights how integrating the host population structure and a solid operational definition of phage species allows us to better appreciate phage diversity and its transmission dynamics.

**IMPORTANCE:**

Much knowledge about bacteriophages has been obtained via genomics and metagenomics over the last decades. However, most studies dealing with prophage diversity have rarely conducted phage species delimitation (aspect 1) and have hardly integrated the population structure of the host (aspect 2). Yet, these two aspects are essential in assessing phage diversity. Here, we implemented an operational definition of phage species (clustering at 95% identity, 90% coverage) and integrated the host’s population structure to understand prophage diversity better. Gathering the most extensive data set of *Acinetobacter baumannii* phages, we show that most prophage species have four or fewer prophages per species, and just five prophage species have more than 100 prophages. Most prophage species have a narrow host range and are geographically restricted; yet, very few have a broad host range being well spread in distant lineages of *A. baumannii*. These few broad host range prophage species are cosmopolitan and the most abundant species. Prophages in the same bacterial genome are very divergent, and prophages can easily be gained and lost within the bacterial lineages. Finally, even with this extensive data set, the prophage diversity has not been fully grasped. This study shows how integrating the host population structure and clustering at the species level allows us to better appreciate phage diversity and its transmission dynamics.

## INTRODUCTION

Bacteriophages, viruses infecting bacteria, are the most abundant genetic entities in the world, with the number of bacteriophages estimated to be 4.8 × 10^31^ (1). Bacteriophages (or phages for short) are major players in the ecology and the evolution of bacterial populations. Considering their life cycle, the vast majority of phages can be divided into lysogenic or lytic types. In the lytic cycle, upon entrance, phages replicate and the bacterial cell is destroyed when virions are released. In the lysogenic cycle, the viral genome is integrated into the bacterial genome and transmitted along with the bacterial genome; these are the so-called prophages. Recently, analyzing over 13,000 bacterial genomes from very different genera, we showed that prophages are found in 75% of bacterial genomes and that human bacterial pathogens are enriched in prophages (2). Contrary to the common belief that prophages are passive genetic elements, they can majorly impact bacterial populations (3). For instance, prophages carrying toxins have been associated with important human diseases such as cholera (4, 5). They have also been associated with the transfer of antibiotic resistance genes in relevant human bacterial pathogens (6, 7). Also, prophages could be important players in the diversification of bacterial populations (8–10), for instance, affecting recombination in the bacterial populations (9, 10). Through lysogeny, phages can be present in diverse microbial communities and disperse along with their bacterial hosts. Over the last decades, several factors have promoted research on phages. On the one hand, the increasing affordability of genome sequencing and the development of bioinformatic programs have significantly impacted bacteriophage research (11). On the other hand, given the current antibiotic resistance crisis, phages have gained much attention as an alternative treatment against bacterial infections (12–14). This is particularly true for important nosocomial pathogens. For instance, several successful phage therapy cases have been reported for very severe *Acinetobacter baumannii* infections (13, 15).

*A. baumannii* is a significant human pathogen. It is a frequent cause of multidrug and even pandrug-resistant infections in many parts of the world. The World Health Organization considers carbapenem-resistant *A. baumannii* as one of the highest-priority bacteria for which novel antibiotics are urgently required (16). Part of the success of *A. baumannii* in the hospital is due to the incredible ability to acquire mobile genetic elements very rapidly (17). In this regard, it has been shown that prophages seem to be a common occurrence in *A. baumannii* genomes (6, 7, 18, 19). Moreover, we and others have shown that prophages can be important agents for transferring virulence and antimicrobial resistance genes in this bacterial species (7, 20, 21). Nonetheless, there are important aspects these studies have not taken into account. These studies did not define “phage species” to conduct their analyses nor did they explicitly take into account the population structure of *A. baumannii*. Importantly, the lack of consideration of these two aspects (phage species delimitation and integrating the host population structure) applies to the majority of phages studied regardless of the bacterial species considered. A few interesting studies have used average nucleotide identity (ANI) analyses to define viral lineages or populations (22–25). However, these studies have focused on phages from bacterial taxa above bacterial species (22, 25) or whole biomes (24). Yet, some recent studies have used ANI to describe novel phages (26–28). Importantly, these studies have barely paid attention to prophages, and, more relevant, the population structure of the bacterium has not been incorporated. Nonetheless, there has been a vibrant attempt to provide guidelines for a consistent and comprehensive virus taxonomy at the level of species and beyond (29).

Here, we gathered the most extensive database ever created for *A. baumannii* phages. In total, more than 4,150 prophage sequences were considered. Furthermore, we not only considered prophages but also incorporated virulent phages publicly available. Importantly, substantial arguments about properly defining virus species have been put forward over the last few years (29–31). In this regard, studies have shown the potential of operationally defining phage species to better understand the physiology and phage–host interaction for *Pseudoalteromonas* phages (22) and the role of recombination as a major evolutionary force in the case of cyanophages (23). Following the recommendations of the Executive Committee of the ITCV and expert virologists (29), we implemented a solid phage species definition (clustering at ≥95% identity and ≥90% coverage) to have an unusual fine resolution of the transmission dynamics of the prophages. Finally, differing from previous studies, we explicitly included the population structure of the host (*A. baumannii*) by considering the sequence type (ST) of the bacterial isolates. Of note, albeit not without issues (32), ST determination under multilocus sequence typing (MLST) has been very useful in understanding the population structure and molecular epidemiology of *A. baumannii* (32–34). Taking into account these two aspects, we analyzed the host and geographic range of *A. baumannii* prophages as well as polylysogeny. Our results show that, on the one hand, most prophage species have a narrow host range and are geographically confined. On the other hand, very few prophage species have a broad host range and are cosmopolitan and very abundant. In addition, polylysogens had very divergent prophages, belonging to different prophage species.

## RESULTS

### The most extensive data set of *A. baumannii* prophages

**Fig 1:**
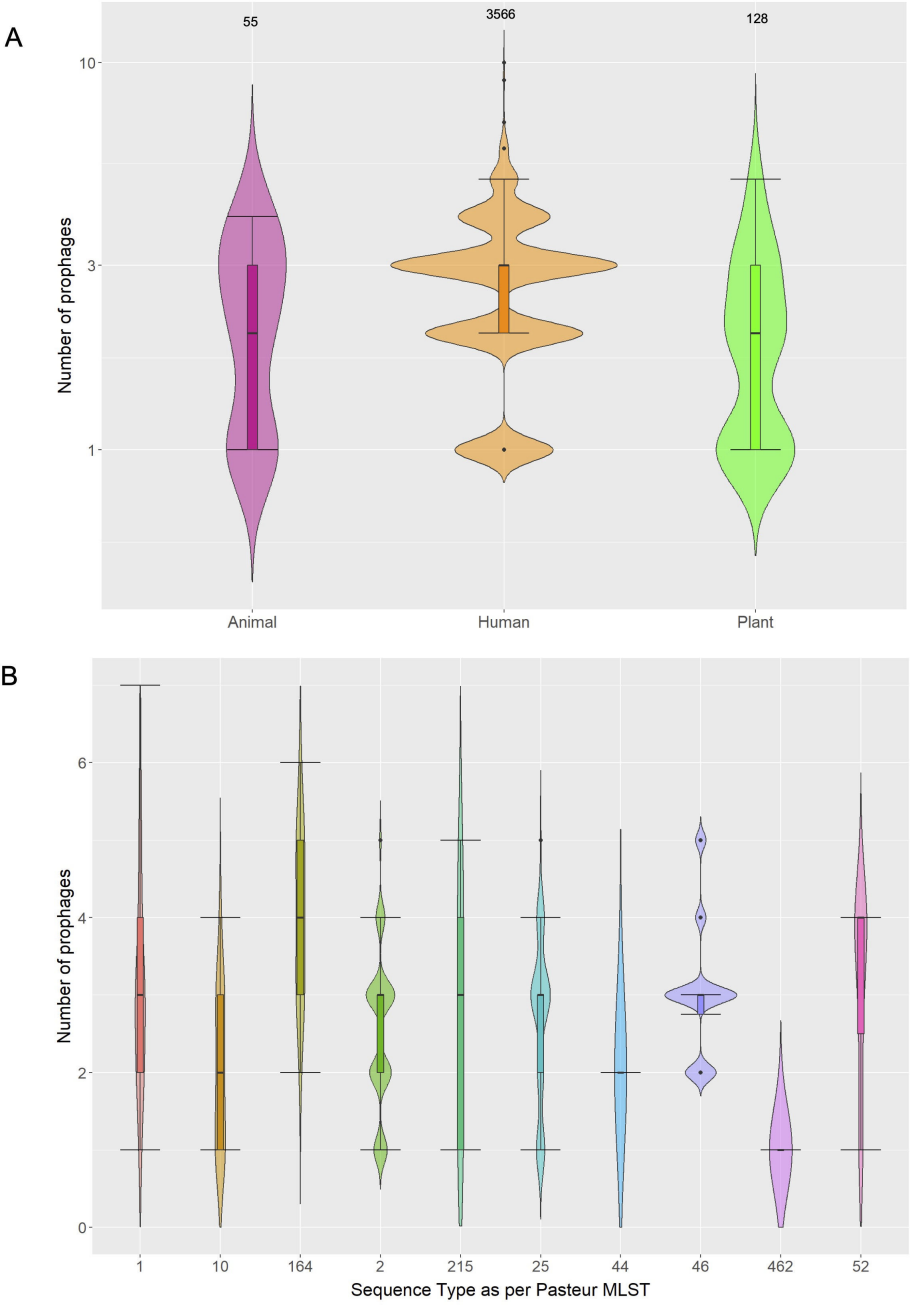
Frequency of prophages. **(A**) Number of prophages in bacterial isolates from different hosts: animal (pink), humans (orange), and plants (green). The numbers on the top of the violin plots indicate the bacterial genomes considered in each case. (B) Number of prophages in bacterial isolates belonging to different STs as per Pasteur MLST. Only the STs with 10 or more bacterial isolates are shown. Each color refers to a different ST.

To have a very comprehensive view of the prophages in *A. baumannii*, we created the most extensive database in terms of *A. baumannii* prophages. We gathered more than 1,600 bacterial genomes from four previous studies (35–38) amounting to 100 STs as per the Pasteur MLST scheme. Of note, these *A. baumannii* genomes come from different hosts, not just humans, and we have studied their genomic epidemiology and population structure previously (35–38). These genomes come from more than 40 countries over four decades. Table S1 lists all the bacterial genomes and their metadata. On these bacterial genomes, we conducted an *in silico* prediction of the prophages and only considered reliable predictions (see Materials and Methods). Only 55 bacterial genomes did not have reliable prophage predictions, and 1,558 bacterial genomes (96%) had reliable predictions. Although initially we had a collection of 20,644 potential prophages, after quality control filtering (see Materials and Methods), we kept 4,152 prophages. We also included the genome sequences of 122 *A*. *baumannii* virulent phages (see Materials and Methods). To the best of our knowledge, this is the most extensive database of prophages ever created for *A. baumannii* or any other ESKAPE (*Enterococcus faecium*, *Staphylococcus aureus*, *Klebsiella pneumoniae*, *Acinetobacter baumannii*, *Pseudomonas aeruginosa*, and *Enterobacter* species) pathogen. Fig. S1 shows the distributions of both prophages and virulent phages in the world, and it can be seen that this data set covers many countries all over the world. Prophages were distributed in 43 countries, whereas virulent phages were present in 15 countries; in total, 46 countries had prophages and/or virulent phages. Yet, some countries had considerably more prophages than others. A relevant aspect compared with previous studies regarding *A. baumannii* (pro)phages is that we included bacterial isolates from humans, animals, and plants. First, we compared the number of prophages in bacterial isolates from different hosts (Fig. 1A) and noted that the number of prophages differs between different hosts (Kruskal–Wallis, *P*-value =2.841*e*−6). Whereas the median is three prophages per bacterial isolate for humans, the median for both animal and plant isolates is slightly over 2. Only four bacterial genomes had eight or more prophages; two belonged to ST79 [an ST mainly found in Latin America (39)], and the other two had no ST assigned. Then, we compared the number of prophages across different STs, thus considering the population structure of *A. baumannii*. Of note, this analysis permits us to study the variation in the number of prophages over much shorter timescales than the timescale that encompasses the whole bacterial species. Figure 1B shows there is considerable variation between the different STs (Kruskal–Wallis, *P*-value =2.8*e*−11) and also within them. Here, we can see that within individual STs (bacterial lineages), prophages are frequently lost and gained. Collectively, the results show that prophages are a very common occurrence in human bacterial isolates and also in non-human bacterial isolates. Furthermore, it seems that prophages are frequently acquired and lost.

### Clustering at the species level unveils lots of diversity

**Fig 2:**
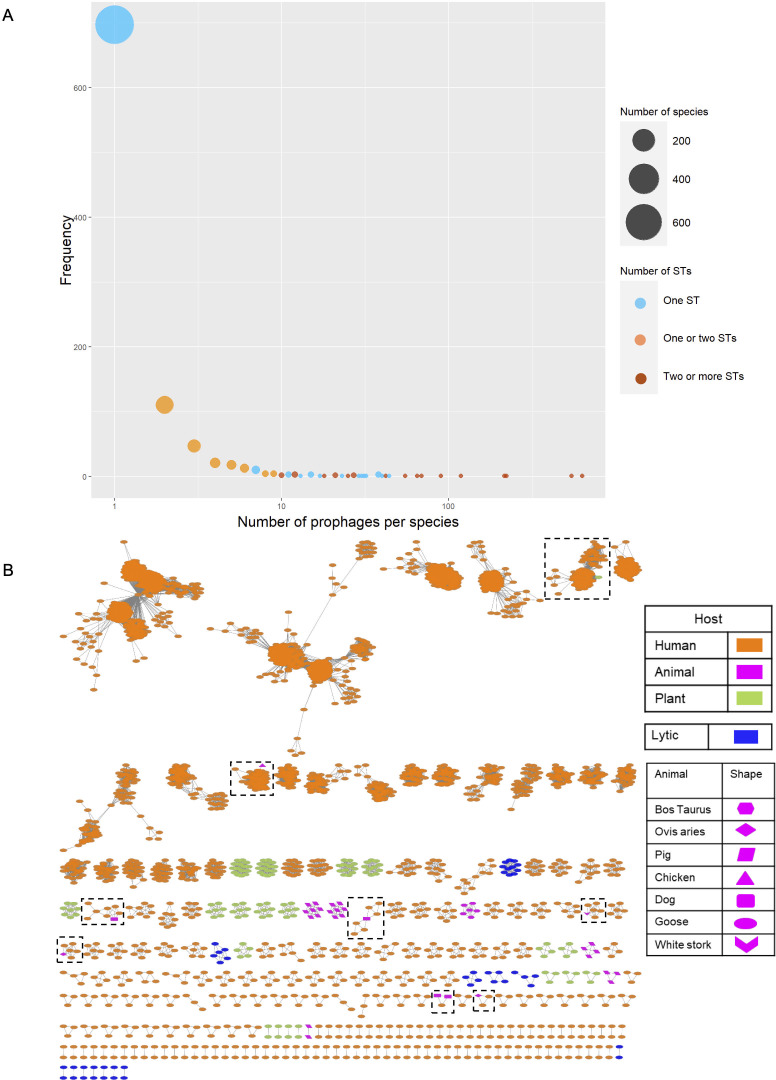
Number of prophage species. **(A**) Frequency of prophage species with different numbers of prophages. The size of the circle (and the *y*-axis) denotes the frequency of prophage species with a given number of sequences (*x*-axis). Blue denotes cases where prophage species were only found in one ST; orange indicates that some prophage species were found in one ST but other species in two STs, whereas dark red shows that prophages were found in two or more STs. (**B**) Network showing the Ab prophage species with two or more sequences per cluster; the nodes are color-coded as per the host (see color key), and shapes in the case of animal hosts show different types of animals. The dashed squares highlight hybrid Ab_PS where prophages came from bacterial isolates from different hosts.

To better understand the prophage diversity at a very fine scale, we conducted phage clustering at the species level (see Materials and Methods). First, we conducted an ANI analysis all versus all. Then, we implemented an operational definition of phage species by clustering at ≥95% identity and ≥90% coverage. The 4,152 prophages were grouped into 963 clusters (from now on prophage species), and the virulent phages formed their clusters (90 groups in total) not containing any prophages. Ab prophage species (Ab_PS) were numbered/named from 1 to 963. To create short names, the acronyms Ab (for *A. baumannii*) and PS (for prophage species) were followed by the number of species; thus, Ab_PS10 refers to “*A. baumannii* prophage species 10.” The frequency distribution of the number of prophages per Ab_PSs was very uneven (see Fig. 2A). Interestingly, 266 Ab_PSs (28%) were proper groups in terms of having two or more prophages, whereas 697 Ab_PSs (72%) were singletons, Ab_PSs just having one prophage (see Fig. 2A). To evaluate if the criteria used for the clustering were too strict (thus inflating the proportion of singletons), we conducted another much lenient clustering, this time lowering the criteria (≥70% identity and ≥70 coverage; see Fig. S2a). In this latest clustering, we also found that the proportion of singletons is around 70%. Furthermore, these two clusterings produce very similar distributions (Kolmogorov–Smirnov test, *D* = 0.10652, *P*-value =0.9449). To see if the 95% identity criterion was problematic (too strict), we plotted all the ANI comparisons between all the prophages that have ≥60% coverage and ≥60% identity. The histogram in Fig. S2B clearly shows that most comparisons (>90% of the data) have identity values higher than 95%. Thus, it seems that our criteria are not too strict.

Then, to better visualize the Ab_PSs and lytic Ab phage species, we constructed a network considering the clustering at the species level (see Fig. 2B). For the sake of clarity, only species having two or more sequences are displayed. From this network, it can be appreciated that Ab_PSs and virulent phage species did not mix at all, meaning that they are well differentiated from one another. It can also be noticed that generally, Ab_PSs in bacterial isolates from different hosts (human, animal, and plants) did not mix as well. For instance, Ab_PSs in bacterial isolates from plants (see green ovals) were only found there and not in bacterial isolates from other hosts, except for just one prophage from a grass isolate that belongs to an Ab_PS with mostly (118/119) prophages from human isolates. This agrees with our initial genomic epidemiology studies of *A. baumannii* (37, 38), where we noted that non-human isolates belong to bacterial lineages not related to the main human international clones (ICs). Yet, there are a few exceptions, where Ab_PSs were found in bacterial isolates from different hosts. Most of these exceptions (7/8) have prophages coming from human and animal bacterial isolates (see dashed squares in Fig. 2B). Thus, prophages in bacterial lineages from different hosts form distinct Ab_PSs.

**Fig 3:**
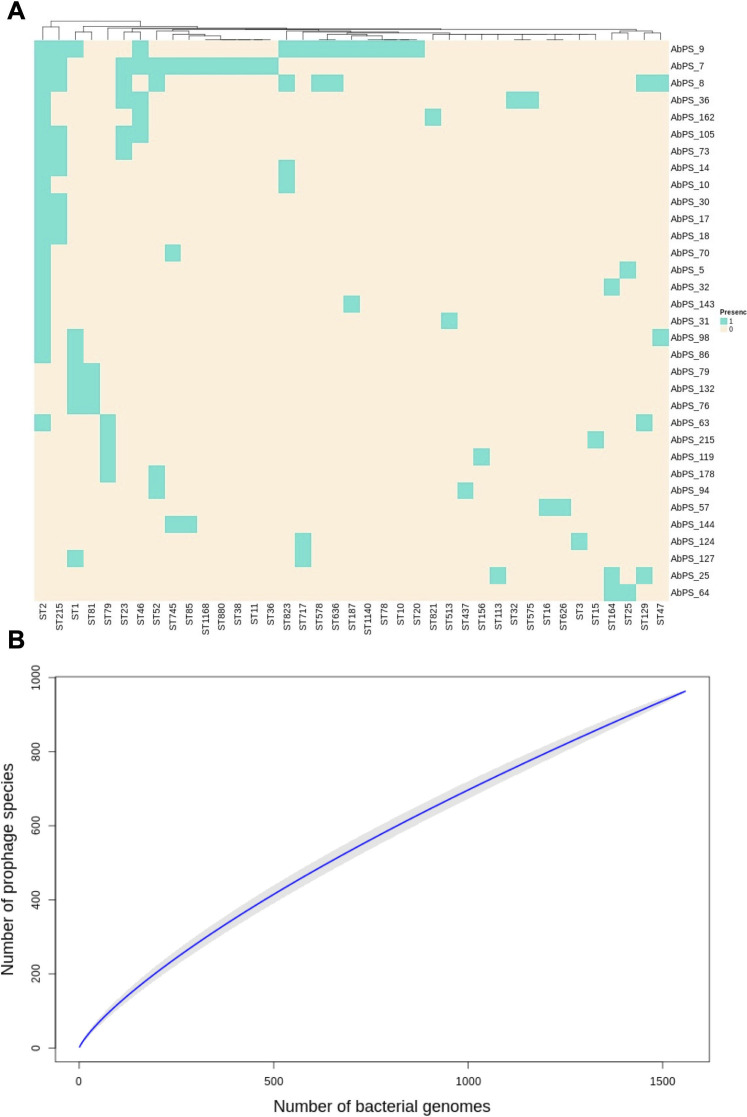
Diversity of prophage species. **(A**) Heat map showing those prophage species that were found in bacterial genomes from two or more STs. To the right, there are the Ab prophage species, and at the bottom, there are the different STs. Blue denotes presence, and cream denotes absence. (**B**) Ab prophage species accumulation curve. The *y*-axis gives the number of prophages as a function of the bacterial genomes used to infer the prophages. The gray area denotes the confidence intervals.

Six hundred one Ab_PSs could be assigned to STs, and 569 of them were found in just one ST. Thus, most prophage species assigned to STs show a narrow host range being present in just one bacterial lineage. On the other hand, there are 33 Ab_PSs found in bacterial genomes from two or more STs (see Fig. 3A; Table S3). Most of these 33 clusters are present in two or three STs; these cases might represent a “medium” (not so narrow) host range. Of note, for many of the clusters present in two or three STs, the average allelic difference between the STs in the cluster is rather high (see Table S3), indicating that the STs are not closely related. Remarkably, three extreme PSs are present in nine or more STs (see Fig. 3A; Table S3). Ab prophage species 7 (Ab_PS7) is found in 12 STs, Ab prophage species 8 (Ab_PS8) is found in 9 STs, and Ab prophage species 9 (Ab_PS9) is found in 13 STs. Of note, in each of these three extreme Ab_PSs, the STs belong to different ICs of *A. baumannii* (IC1, IC2, IC8, and IC9) and, thus, are well disseminated within the whole *A. baumannii* species. Given the vast number of bacterial genomes considered and the respective prophages inferred from them, we wanted to know if we have sampled the vast majority of prophages present in *A. baumannii*. Thus, we ran a species accumulation curve, which is shown in Fig. 3B. The curve clearly shows that we have not sampled the whole diversity of prophages. As one can see, the curve does not level off; on the contrary, the slope in the last section of the curve is still very steep. This squares well with the fact that most of the Ab_PSs are singletons present in just one bacterial genome. Taken together, these results show that most Ab_PSs have a narrow host range, yet there are several exceptions with a broader host range. Additionally, even with this extensive data set, the whole prophage diversity has not been fully grasped.

### A few but very abundant cosmopolitan prophages

**Fig 4:**
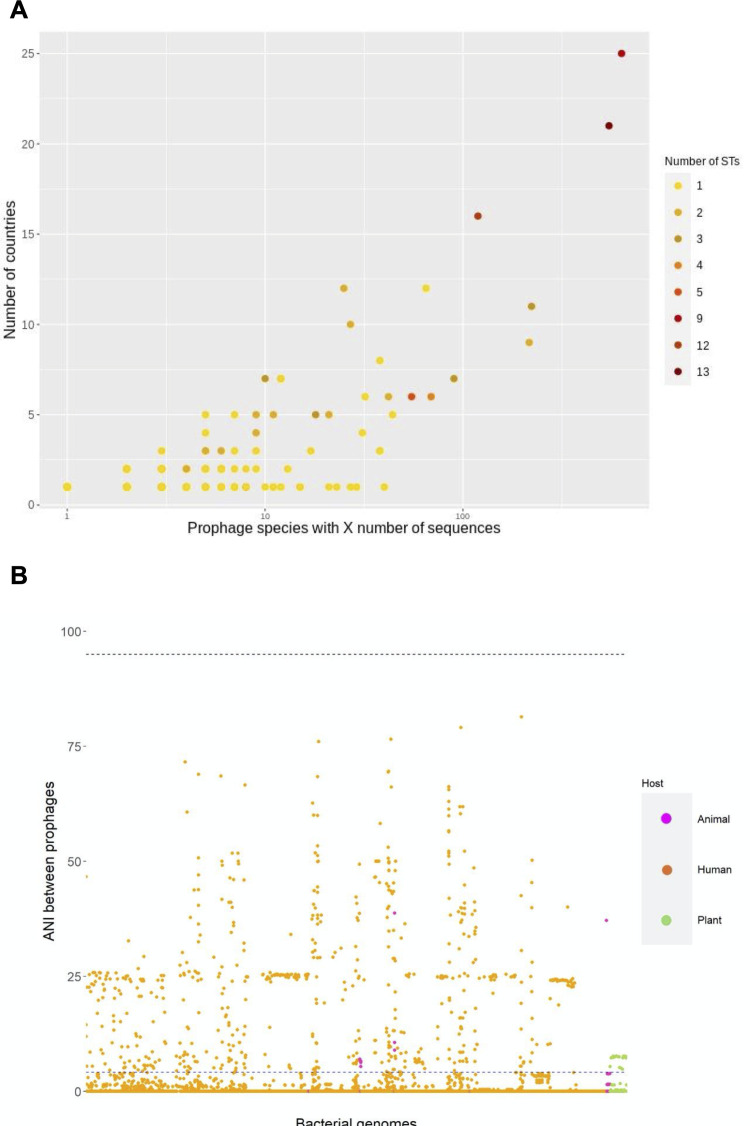
Prophage geographic expansion and polylysogeny. **(A**) Prophage species with *X* number of prophages (*x*-axis) found in different countries (*y*-axis). The color of the dot shows how many STs the prophages are found. (**B**) Similarity of prophages within the same bacterial genome. ANI values between the prophages in individual bacterial genomes. Color code as follows: animal (pink), humans (orange), and plants (green). The black dashed line denotes an ANI value of 95%, whereas the blue dashed line shows the mean of all the ANI values (~4%).

Then, we evaluated the geographic dispersion of the Ab_PSs. Most of the Ab_PSs (90%) are presented in just one or two countries, and few are present in three or more countries. Figure 4A shows the country dispersion and ST distribution for the Ab_PSs. It shows that Ab_PSs present in one, two, and three STs can be present in 1–11 countries (see Fig. 4A). Notably, there were many Ab_PSs with 10 or fewer prophages that were present in five or fewer countries (see bottom right section in Fig. 4A). To see if the Ab_PS singletons may affect this drastically, we analyzed the data with (the whole data) and just the proper groups. We noted that *Q*_2_ (median) was one country for both of them. Furthermore, the upper bounds as per Tukey’s method were very similar (two countries in the whole data set and two countries in the data just considering the proper groups). Thus, the singletons do not significantly bias our findings. Remarkably, we noted three Ab_PS outliers present in many countries (>15) that were also the Ab_PSs most widely distributed in terms of STs (see the top right corner in Fig. 4A). These are the previously mentioned Ab_PS7, Ab_PS8, and Ab_PS9 (see the section above). Figure S3 shows the geographic distribution of Ab_PS8 and Ab_PS9, the most extensively distributed cases. These two Ab_PSs were present in countries from several different continents. It is important to highlight that Ab_PS8 and Ab_PS9 are not only the most widely distributed but also the most abundant Ab_PSs. Whereas Ab_PS8 had 633 prophages (the most abundant), Ab_PS9 had 547 prophages; actually, these two Ab_PSs account for 28% of the total number of prophages. Collectively, these results show that most Ab_PSs are geographically restricted (present in few countries), yet a few Ab_PSs are globally distributed and have the biggest number of prophages.

### Polylysogeny involves highly divergent prophages

The level of polylysogeny changes very fast. If one looks at individual STs, it is clear that the number of prophages changes considerably from one bacterial genome to another (see Fig. 1B). For instance, the bacterial genomes in ST1 can have from one to seven prophages per genome. This high level of variation applies to most of the STs considered. Then, to know how different the prophages found in the same bacterial genome are, we computed the ANI between prophages within individual bacterial genomes (see Fig. 4B). This figure shows that prophages within the same genome are highly divergent. Note that for most of the prophage comparisons within the same bacterial genome, the highest ANI values are below 50%, and there are just a few cases where the highest ANI value is above 50%. The mean ANI value for all the intra-bacterial genome prophage comparisons is 0.0403 (standard deviation = 0.1077). We did not find a single case where prophages from the same prophage species were found in the same bacterial genome, which is expected, given the superinfection exclusion mechanism (40–42). Thus, these data show that the prophages present in a bacterial genome are from very well-diverged Ab_PSs.

## DISCUSSION

The study of phages has been hampered by the shortfall in an extensively agreed system to classify them (or all viruses for that matter) into natural clusters, representing ecological and evolutionary cohesive units. This has been changing over the last few years, and some studies have conducted phage species delimitation (22–25). However, proper species delimitation is still not commonplace when analyzing phage diversity. Furthermore, the inclusion of the population structure of the host (the bacterium) is another major shortfall that hardly any study has taken into account. Here, we not only conducted a solid operational definition of phage species for prophages found in *A. baumannii* but also incorporated the population structure of the bacterium to characterize the diversity of prophages.

Over the last few years, some studies have analyzed prophages in *A. baumannii* (6, 7, 18, 19). Our study considerably supersedes previous studies in at least three important points. First, we cluster the prophages at the species level. This gave us a coherent and very fine resolution hardly seen in previous population genomic studies of phages. Due to this, we could better grasp the prophage diversity within this important human pathogen (read the paragraph below). Second, we explicitly incorporate the population structure of the host (*A. baumannii*) by considering the STs to which the bacterial isolates belong. This is very relevant. For instance, if you have a phage that infects two bacterial strains, you can have that those bacterial strains are very closely related (same lineage, same ST), but you can also have that those bacterial strains are not closely related belonging to different lineages (different STs). In this regard, typically, when evaluating the host range of phages, several bacterial strains are considered; however, hardly anyone pays attention to how closely related these bacterial strains are. Finally, this is the most extensive study considering over 1,500 bacterial genomes and more than 4,150 prophages; previous studies have considered bacterial genomes for their predictions in the order of hundreds (6, 7, 18) but not in the order of thousands. Notably, we considered not only human bacterial isolates but also isolates from plants and animals.

Our clustering of prophages at the species level unveiled some important trends. First, we found almost 1,000 species of phages in *A. baumannii* illustrating the phage diversity that can infect a single bacterial species. Second, most of the Ab prophage species (72%) are just composed of one prophage and are bacterial genome-specific, whereas 28% of the clusters have two or more prophages. However, even in this 28% of the clusters, most of them are in bacterial genomes from the same ST, thus having a narrow host range. Yet, we also found some significant exceptions, as a few Ab_PSs were found in several STs. These exceptions show that in some instances, the host range of some prophages is not narrow at all. This squares up with recent studies showing that broad host range phages are not unusual and that some environments have an assortment of broad and narrow host range phages (43). Another major finding is that most of the Ab_PSs are geographically restricted being present in just one or two countries. Contrary to this, a few cosmopolitan Ab_PSs were found, and these are by far the most abundant Ab_PSs representing 28% of the total number of prophages. Another trend is that Ab_PSs are well differentiated from the virulent phage species. Finally, our accumulation curve analysis shows that, even with the massive amount of prophages and bacterial genomes here considered, we are far from sampling the whole diversity of prophages. Thus, many more prophages are still to be found. Of note, other studies have also found that phages can be defined in coherent ecological and evolutionary units at >95% identity (22, 23). For instance, a study focusing on different phages (*Pseudoalteromonas* phages) defined phage species—they used “phage OTU” rather than species, though—using a cutoff value of 95% identity (considering at least 80% of the genome) and showed that this strategy is very useful for analyzing the host range and phage–host interactions (22). It is important to note that the actual number of prophages per phage species is bound to change as the databases of bacterial genomes and virulent phages grow over time. For instance, some singletons might become proper groups with two or more sequences. However, we think that the general trends might persist; thus, even if previous singletons become proper groups, they might be still present in one ST and single countries. We acknowledge that an important limitation of our study is that most bacterial genome samples come from not very many countries and mostly humans. Ideally, sampling should come from most countries and different organisms not mainly humans.

Our analyses of the prophages coexisting in a bacterial genome (polylysogeny) show that the typical *A. baumannii* genome has between 2 and 3 prophages, yet there are some extreme cases where 8, 9, and even 10 prophages were found. These figures (two and three prophages) differ from estimates from previous studies that found many more prophages per bacterial genome (7, 18). We think that this is due to the fact that we used stricter criteria to infer the prophages (see Materials and Methods). Our ANI analysis shows that these coexisting prophages belong to highly divergent phage species. Considering that genomic epidemiology studies of the bacterial host have shown that several lineages of *A. baumannii* are cocirculating in different settings (36, 37), this will imply that several phage species dwell in the locales where *A. baumannii* is circulating. On the other hand, there is considerable variation in the number of prophages per bacterial genome in bacterial lineages (STs). Also, bacterial isolates from different hosts vary in the number of prophages. Bacterial isolates from plants seem to have fewer prophages than bacterial genomes from humans. Whereas many prophage species with very low prevalence (singletons and prophage species with few prophages) have been recently acquired, the more distributed prophage species could have been due to ancient integrations (with subsequent losses in some cases) or have been acquired multiple times.

In summary, our study shows that most prophage species have a narrow host range and are geographically restricted. Nonetheless, very few prophage species have a broad host range and are cosmopolitan and very abundant. Also, polylysogens have different (very divergent) prophage species. In a broader context, this work shows that a phage species definition is of paramount importance to understanding the transmission dynamics of the phages properly.

## MATERIALS AND METHODS

### Prophage prediction and lytic phages

We employed four previous data sets (35–38) to construct one of the largest genome databases for *A. baumannii* prophages. Table S1 lists all the bacterial genomes considered, their accession numbers, and all the metadata associated with them. In total, 1,613 genomes were considered. Notably, this database has not only human but also animal and plant isolates. *In silico* prophage prediction was conducted on the bacterial genomes using VirSorter2 v2.2.3 (44), considering the default parameters. We verified the prophage signals employing CheckV v1.0.1 (45). For downstream analyses, we only kept prophages that show >50% completeness and that were inferred from contigs having at least 30,000 bp. Contigs with more than one prophage signal were checked and separated manually. Table S4 lists all the prophages included in the analyses. We also downloaded the reported *A. baumannii* phage genomes from NCBI. We used Bacphlip (46) and PhageAI (47) to infer the life cycle for these phage genomes, and only those assigned as lytic phages by both programs were used for downstream analyses. Table S2 provides accession numbers as well as the metadata of these phages, including the genus, family, and life cycle.

### Prophage species, host range, geographical dispersion, and polylysogeny

Our operational phage species definition was as follows. Considering both prophages and lytic phages, we conducted an all-versus-all ANI analysis via Pyani v0.2.11 (48). The prophage signals were clustered at the species level, that is, >95% identity and >90% coverage, using an in-house PERL script. The script takes the matrix of all-versus-all ANI values and creates clusters that are >95% identity and >90% coverage considering a paraclique clustering strategy (49, 50). The results were visualized as a network constructed with Cytoscape v.3.8 (51). Once the Ab prophage species (Ab_PSs) were determined, we conducted a host range analysis and also determined the geographical distribution of those Ab_PSs. To infer the host range of the prophage species, we recorded the different numbers of sequence types (STs) from the bacterial genomes where the prophages reside. In previous works (35–38), we have assigned STs to every bacterial genome when possible. To analyze the geographical dispersion, we used all the prophage species that came from bacterial genomes that have a country assigned to them (see the section below). As for the polylysogeny analyses, for each bacterial genome, the total number of prophages was counted. Then, all the prophages within the same bacterial genomes were compared against each other by running an ANI analysis between them.

### Metadata, statistical analysis, and plots

All the metadata (country, host of the bacteria, etc.) about the prophage were extracted from the information of the bacterial genomes on which the prophages were predicted. This information was extracted from the BioSample records of the bacterial genomes, which were downloaded from NCBI. The species accumulation was run using the vegan library v2.6-4 in R (52). For the species accumulation curve, we used the “specaccum” function with the “rarefaction” method. For the comparison of the number of prophages in bacterial isolates from different hosts, only bacterial genomes with proper host assignation were considered. Whereas for the comparison of the number of prophages per STs, only bacterial genomes with ST assignation were considered. All the statistical analyses were conducted in R. Violin, boxplots, and bar plots were also produced using R.

## Data Availability

All the bacterial genomes used in this study are listed in Table S1. This table provides the BioSample numbers for all of them.

## SUPPLEMENTAL MATERIAL

Supplemental Figures mbio.02377-24-s0001.pdf Figures S1 to S3.

Table S1 mbio.02377-24-s0002.xlsx All the bacterial genomes and their metadata.

Table S2 mbio.02377-24-s0003.xlsx Virulent phages and their metadata.

Table S3 mbio.02377-24-s0004.docx Prophage species present in bacterial genomes from 2 or more STs.

Table S4 mbio.02377-24-s0005.xlsx Prophage predictions and their coordinates.

ASM does not own the copyrights to Supplemental Material that may be linked to, or accessed through, an article. The authors have granted ASM a non-exclusive, world-wide license to publish the Supplemental Material files. Please contact the corresponding author directly for reuse.
