# Supplementary material for: Host population structure and species resolution reveal prophage transmission dynamics
Source: mBio. 2024 Sep 24;15(10):e02377-24. doi: 10.1128/mbio.02377-24 (PMC11481511; doi:10.1128/mbio.02377-24)
Supplement: Supplemental Figures — Figures S1 to S3. [file mbio.02377-24-s0001.pdf]

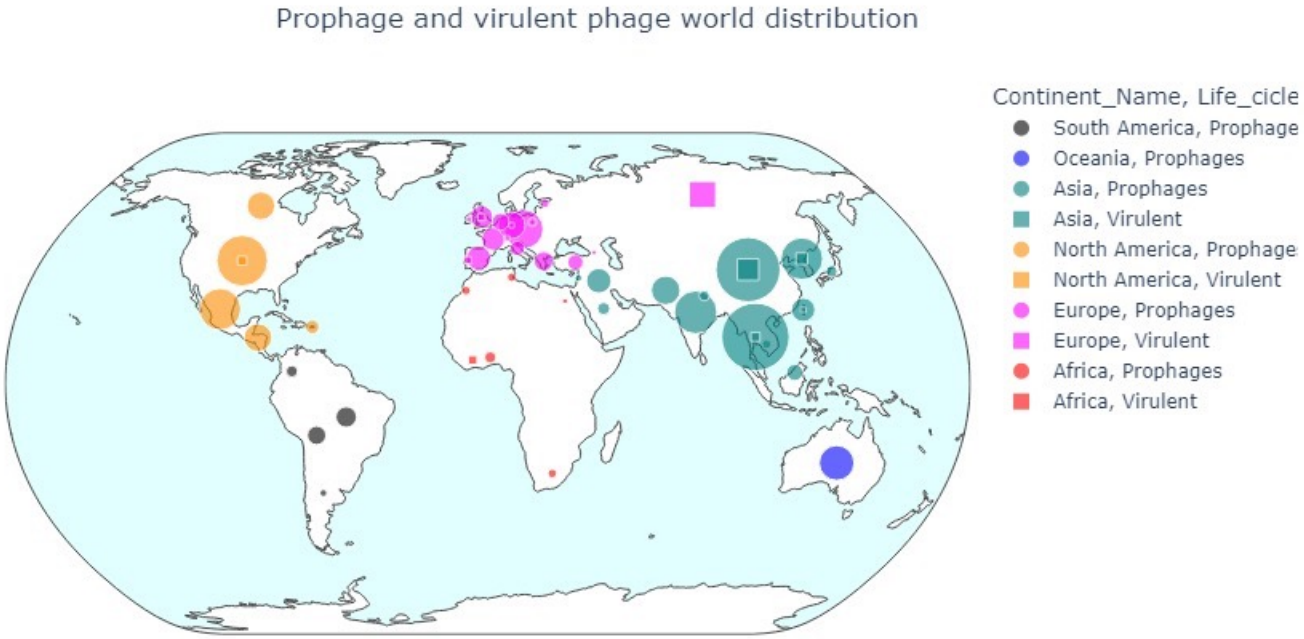

Supplementary Figure 1
World distribution of prophages and virulent phages. Prophages are shown in circles and virulent phages are in squares. The size of the circle/square gives the number of cases.

Panel A)

Panel B)

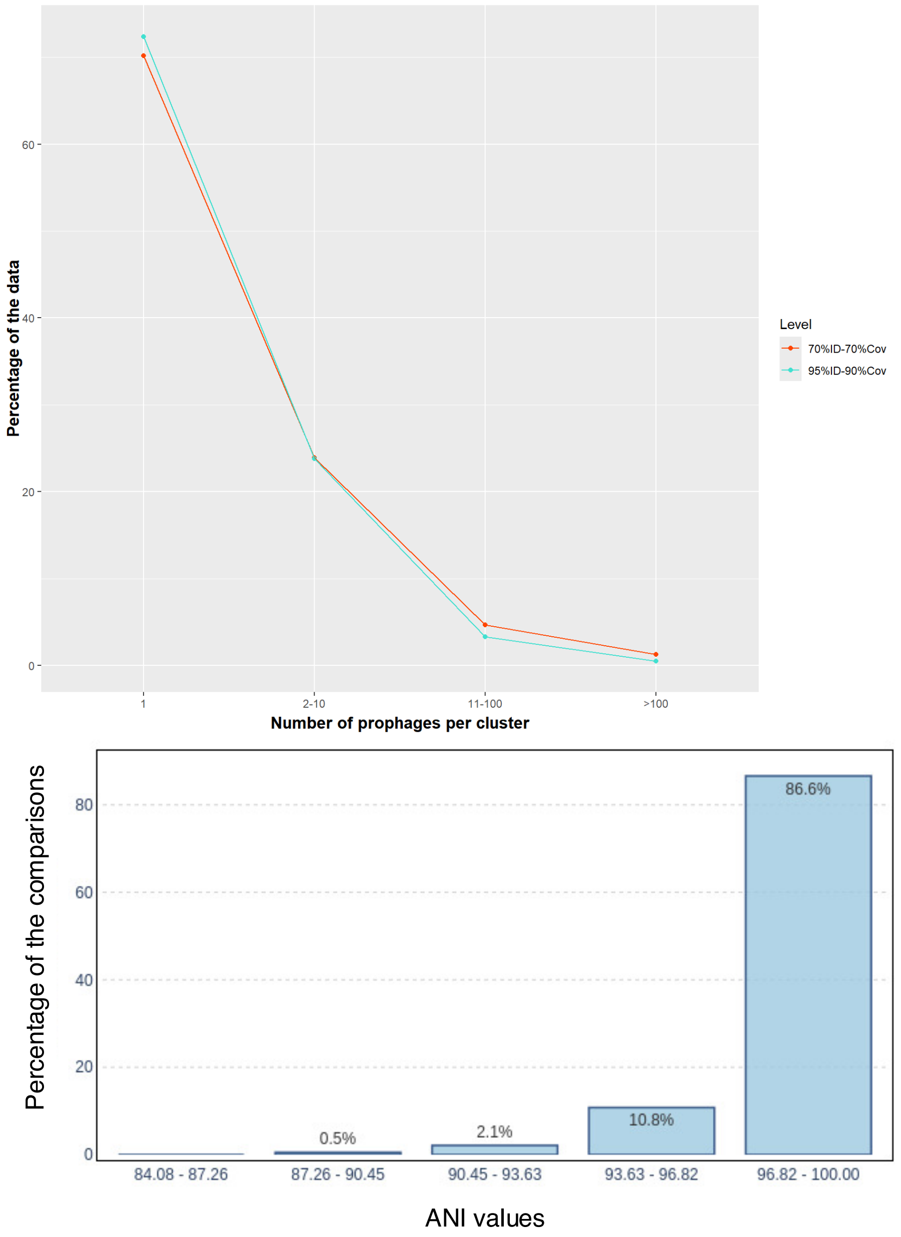

Supplementary Figure 2

Panel A) Distribution comparison of the clustering at ≥ 95% identity and ≥ 90 coverage, and ≥ 70% identity and ≥ 70 coverage. Panel B) Histogram of all the ANI comparisons between all the prophages that have ≥ 60% coverage and ≥ 60% identity.

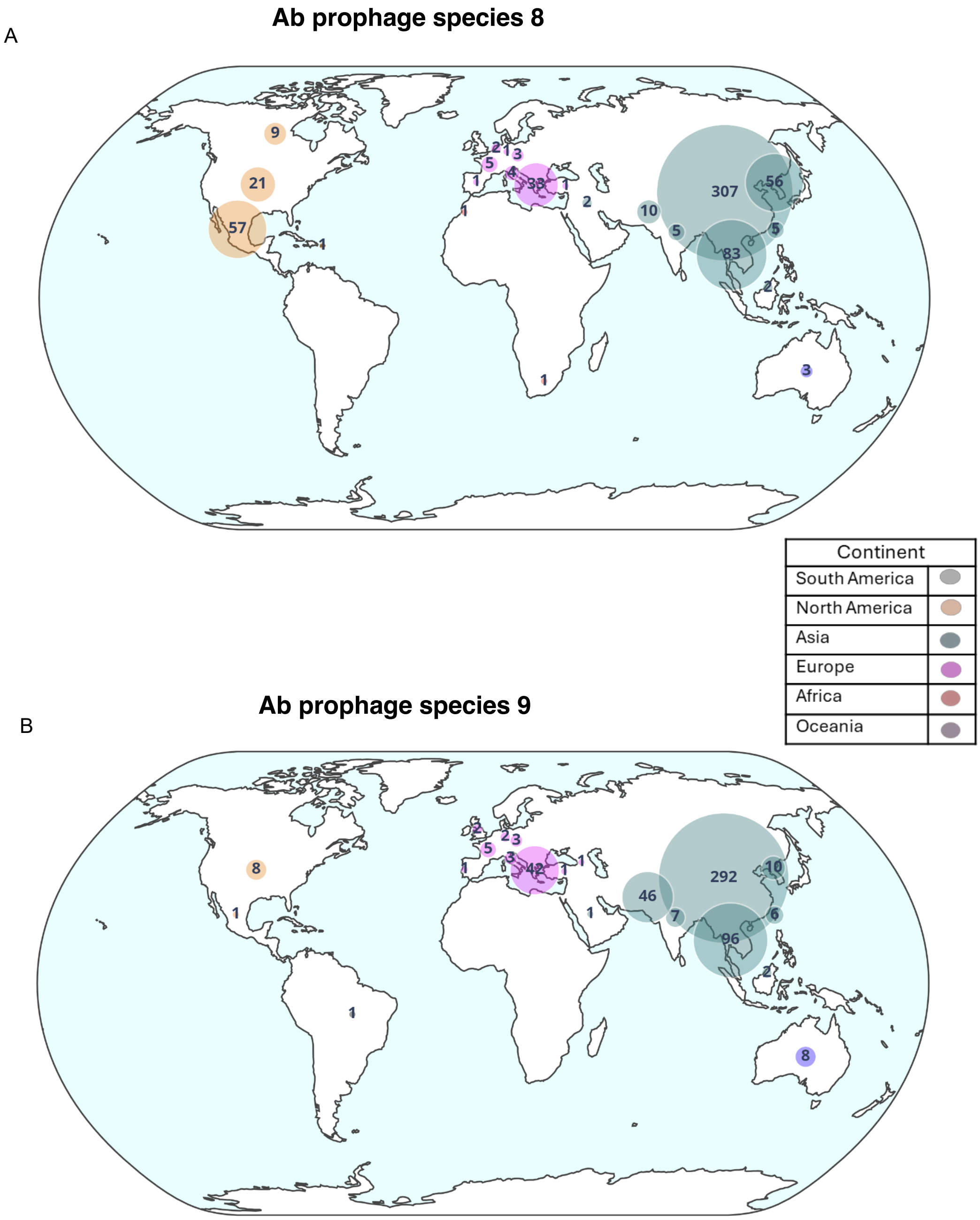

Supplementary Figure 3
Geographic distribution of Ab prophage species 8 (Panel A) and 9 (Panel B).
