# Supplementary material for: Host population structure and species resolution reveal prophage transmission dynamics
Source: mBio. 2024 Sep 24;15(10):e02377-24. doi: 10.1128/mbio.02377-24 (PMC11481511; doi:10.1128/mbio.02377-24)
Supplement: Table S3 — Prophage species present in bacterial genomes from 2 or more STs. [file mbio.02377-24-s0004.docx]

**Supplementary Table 3**

Prophage species present in bacterial genomes from 2 or more STs

| **Ab Prophage Species.** | **Sequence Type*** | **Number of STs** | **Average allelic difference between STs** |
| --- | --- | --- | --- |
| Ab_PS-10 | ST2, ST823 | 2 | 14.28% |
| Ab_PS-105 | ST2, ST23, ST46, ST215 | 4 | 85.71% |
| Ab_PS-119 | ST79, ST156 | 2 | 14.28% |
| Ab_PS-124 | ST3, ST717 | 2 | 85.71% |
| Ab_PS-127 | ST1, ST717 | 2 | 14.28% |
| Ab_PS-132 | ST1, ST81 | 2 | 14.28% |
| Ab_PS-14 | ST2, ST215, ST823 | 3 | 38% |
| Ab_PS-143 | ST2, ST187 | 2 | 14.28% |
| Ab_PS-144 | ST85, ST745 | 2 | 85.71% |
| Ab_PS-162 | ST2, ST46,ST821 | 3 | 64.28% |
| Ab_PS-17 | ST2, ST215 | 2 | 42.85% |
| Ab_PS-178 | ST52, ST79 | 2 | 57% |
| Ab_PS-18 | ST2, ST215 | 2 | 42.85% |
| Ab_PS-215 | ST15, ST79 | 2 | 85.71% |
| Ab_PS-25 | ST113, ST129, ST164 | 3 | 71.42% |
| Ab_PS-30 | ST2, ST215 | 2 | 42.85% |
| Ab_PS-31 | ST2, ST513 | 2 | 85.71% |
| Ab_PS-32 | ST2, ST164 | 2 | 85.71% |
| Ab_PS-36 | ST2, ST23, ST32, ST46, ST575 | 5 | 77% |
| Ab_PS-5 | ST2, ST25 | 2 | 71.42% |
| Ab_PS-57 | ST16, ST626 | 2 | 57.14% |
| Ab_PS-63 | ST2, ST79, ST129 | 3 | 52.38% |
| Ab_PS-64 | ST25, ST164 | 2 | 71.42% |
| Ab_PS-7 | ST2, ST11, ST23, ST36, ST38, ST46, ST52, ST85, ST215, ST745, ST880, ST1168 | 12 | 74.45% |
| Ab_PS-70 | ST2, ST745 | 2 | 14.28% |
| Ab_PS-73 | ST2, ST23, ST215 | 3 | 80.95% |
| Ab_PS-76 | ST1, ST81 | 2 | 14.28% |
| Ab_PS-79 | ST1, ST81 | 2 | 14.28% |
| Ab_PS-8 | ST2, ST23, ST47, ST52, ST129, ST215, ST578, ST636, ST823 | 9 | 69.84% |
| Ab_PS-86 | ST1, ST2 | 2 | 100.00% |
| Ab_PS-9 | ST1, ST2, ST10, ST20, ST46, ST78, ST187, ST215, ST578, ST636, ST717, ST823, ST1140 | 13 | 77.83% |
| Ab_PS-94 | ST52, ST437 | 2 | 57.14% |
| Ab_PS-98 | ST1, ST2, ST47 | 3 | 71.42% |

* STs as per Pasteur MLST
